# 17547 Transitions of Care among Patients with Diabetes in the Deep South: Factors Associated with Hospital Readmissions

**DOI:** 10.1017/cts.2021.706

**Published:** 2021-03-30

**Authors:** Cassidi C. McDaniel, Chiahung Chou

**Affiliations:** Auburn University Harrison School of Pharmacy

## Abstract

ABSTRACT IMPACT: Because diabetes disproportionately affects residents in the Deep South, identifying factors increasing the risk of hospital readmissions unique to this population can translate to tailored interventions and strategies to improve transitions of care and patients’ health outcomes. OBJECTIVES/GOALS: Patients with diabetes (PWD) are susceptible to hospital readmissions due to inadequate transitions of care (TOC). To better understand how to improve TOC, the objective of the study is to identify factors associated with readmissions among PWD in Alabama disproportionally affected by diabetes. METHODS/STUDY POPULATION: This retrospective cohort study utilizes electronic health record data from an urban health system in Alabama. The study population includes adults (≥18 years old) diagnosed with diabetes who were hospitalized between 2016 and 2020. Women who are pregnant during hospitalization or diagnosed with gestational diabetes are excluded. Patient’s index hospitalization is identified with a 3-month washout period preceding admission. The primary outcome is all-cause 30-day readmission. Characteristics are compared between patients with and without readmissions. Factors significantly associated with readmissions are identified with multiple logistic regression, adjusted for potential confounders. RESULTS/ANTICIPATED RESULTS: The sample size is expected to be around 30,000 individual PWD. Anticipated results include estimation of the all-cause 30-day readmission rate experienced by the PWD in Alabama. It is expected that various factors will be associated with either higher or lower odds of readmission, interpreted via odds ratios and 95% confidence intervals. Factors investigated are driven by previously identified risk factors of readmission from the literature, including but not limited to sociodemographic variables, lab values (A1C, glucose, serum albumin, serum sodium, etc.), vital signs (blood pressure), comorbidities, medications, length of stay, insurance coverage, geographic location, and social history. DISCUSSION/SIGNIFICANCE OF FINDINGS: Findings will establish evidence-based knowledge about TOC for PWD in the Deep South, specifically Alabama. Identifying factors associated with readmissions among PWD in Alabama will inform TOC intervention studies tailored to populations in the Deep South to effectively mitigate readmissions.

